# Dendritic Cell Vaccination in Pediatric Gliomas: Lessons Learnt and Future Perspectives

**DOI:** 10.3389/fped.2013.00012

**Published:** 2013-06-10

**Authors:** Matthias Eyrich, Johannes Rachor, Susanne C. Schreiber, Matthias Wölfl, Paul G. Schlegel

**Affiliations:** ^1^Department of Pediatric Oncology, University Children’s Hospital, University of WürzburgWürzburg, Germany

**Keywords:** pediatrics, malignant glioma, DC vaccination, clinical trial, immunotherapy

## Abstract

Immunotherapy of malignant gliomas with autologous dendritic cells (DCs) in addition to surgery and radiochemotherapy has been a focus of intense research during the past decade. Since both children and adults are affected by this highly aggressive brain tumor, 10–15% of the several hundred vaccinated patients represent children, making pediatric glioma patients the largest uniform pediatric vaccination cohort so far. In general, DC vaccination in malignant gliomas has been shown to be safe and several studies with a non-vaccinated control group could clearly demonstrate a survival benefit for the vaccinated patients. Interestingly, children and adolescents below 21 years of age seem to benefit even more than adult patients. This review summarizes the findings of the 25 clinical trials published so far and gives a perspective how DC vaccination could be implemented as part of multimodal therapeutic strategies in the near future.

## Introduction

Since the earliest descriptions of dendritic cells (DCs) in skin (1) and later also in lymph nodes (2), their pivotal role in the regulation of immune responses has been recognized. It was primarily in histopathological analyses of easy accessible skin tumors that indicated a close interaction of skin DCs and T helper cells (3). These data led to groundbreaking murine studies showing that DCs pulsed with tumor-extracts can significantly delay cancer growth (4), result in humoral and cellular immune responses (5), and confer *in vivo* resistance to tumor challenge (6). Quite rapidly, these findings have been translated into clinical studies, one of the first clinical trials in patients with follicular B-cell lymphoma reported no humoral responses but increased cellular proliferation in 4/4 treated patients and clinical responses of various degree in 3/4 (7). Thousands of articles have been published since then (a recent PubMed search with the keywords “DCs and vaccination and tumor” provides 2348 articles), however, results from up-to-date clinical trials still yield no superior results than in the earliest studies. Although this seems daunting at a first glance, many important lessons have been learnt from these trials.

Dendritic cell vaccination has been performed targeting many different tumor entities, the majority of which belonging to types of cancer typically occurring in adults (melanoma, renal cell carcinoma, breast, colon, and prostate cancer). Malignant glioma has also gained considerable attention in the field, and this tumor is a tumor entity relevant also in pediatric oncology. Even with most recent multimodal treatment strategies, the majority of children will relapse within 5 years and die of progressive disease (8). Thus, this review focuses on insights, advances, and translational aspects in the field of DC vaccination studies for malignant gliomas.

## Current State of the Art

Several murine models using vaccination with DCs in malignant glioma models have shown that this approach holds considerable promise in treating this highly aggressive brain tumor (9, 10). Moreover, there is any evidence that vaccination with DC, even when using whole brain lysate as antigen source, may break tolerance to CNS epitopes (11, 12). This may be explained by the lifespan of mature DC in peripheral lymphoid organs, which is probably only 1–2 to a maximum of 9 days (13), thereby limiting contact time and potential interactions with naïve T cells. Mature DCs are not capable of dividing or self-sustaining anymore, and their intrinsically activated apoptotic pathways have been shown to be pivotal for the prevention of autoimmunity (14, 15). However, despite the short shelf life of migratory DCs, antigens from injected DCs can be transferred to endogenous DCs from host tissue possibly prolonging and enhancing presentation of tumor antigens. In contrast to migratory DCs, these endogenous, tissue-resident DCs have a significantly extended lifespan (13). This process of antigen transfer has been shown to be important for full activation of CD8^+^ T-cell responses and requires an additional, simultaneous TLR activation stimulus (16, 17). Another interesting observation pointed toward the role of oligodendrocytes in preventing CNS-autoimmunity: these intraparenchymal, CNS-resident cells are highly effective in purging the peripheral repertoire of autoreactive T cells (18). Whether this mechanism also applies to tumor associated CNS-antigens remains to be determined.

After intradermal injection, DCs migrate to the next draining lymph nodes (dLN), where the first cells already appear after 30 min with a maximum of DC influx after 48 h (19, 20). Thereafter, numbers of antigen-loaded DCs sharply decline due to apoptosis and become undetectable with conventional imaging within 6 days. With more sensitive genetic marking techniques traces of migratory DCs (<1%) have been detected up to 14 days post-injection (21). Skin-derived DC subsets which repopulate the dLN more continuously (19, 20) as well as LN-resident DCs (22) probably enforce and prolong this antigen-presentation beyond the lifespan of injected, migratory DCs. Several studies have analyzed the body distribution of DCs after intradermal, subcutaneous, or intravenous injection. In humans, significantly more DCs reach the dLN after intradermal than after subcutaneous injection (23–26), so that intradermal application has become the standard delivery route in most trials. However, current estimations from nuclear imaging studies suggest that only 2–4% of injected DCs succeed to migrate to the dLN (27).

Cytotoxic T lymphocytes (CTLs) which have been successfully activated by a tumor antigen presenting DC have to be imprinted with a specific signature of adhesion molecules in order to be able to traffic to the tumor site in the CNS. For CNS-specific homing, the expression of the integrin VLA-4 (a heterodimer of α4, CD49d and β1, CD29) seems to be particularly important (28), although other molecules such as CXCR3 play a role as well (29). The capacity of a DC to induce VLA-4 expression was dependent on the site where the DC picked up the antigen (30) and VLA-4 expression could be inhibited by IL-4 (31). Quite unexpectedly, a recent paper demonstrated that the effectiveness of a DC vaccine was inversely correlated to the proximity of the injection site to the tumor (32). Whether these findings have a clinical relevance and what the specific requirements for an *in vitro*-matured DC are to induce VLA-4^+^ CTLs *in vivo* remains to be determined in future investigations.

To the best of our knowledge, based on an extended literature search, so far there are 25 published articles on DC vaccination in human glioma patients (total of 414 patients, among those approximately 50 children) have been published so far (Table S1 in Supplementary Material). Of these, two were case reports (33, 34), the others single-center phase I/II studies, four publications included a control group without DC vaccination (35–38). Most studies focused on feasibility and safety, and indeed there is now convincing evidence that DC vaccination is safe even in brain tumor patients, since no major (>NCI-CTC grade 2) or dose-limiting toxicity has been reported so far. Initial concerns that the use of brain-derived antigens for vaccination could induce autoimmunity like in melanoma (39, 40) could not be confirmed. Several studies analyzed the impact of injected cell dose on outcome (41–45). There seems to be no correlation between the number of injected DCs and the clinical or immunological responses, in one study patients receiving lower dosages of DCs (1 × 10^6^ DCs/vaccine) even had a better overall survival (OS) than cohorts getting higher numbers of injected cells (44). Cell numbers of >5 × 10^7^ DCs/vaccine simply seem to be unfeasible, because such high numbers of mature DCs can only be generated in a limited number of patients (42). A shorter interval especially between the first four vaccines seemed to be beneficial (46), in contrast, the optimal duration of vaccine therapy is completely unclear. Some authors favor a continued vaccination regimen in order to maintain an induced immune response (47), however, no valid data in this respect is available so far. One important consideration associated with prolonged exposure to antigen is the induction of tolerance. Although never observed in high-grade gliomas or DC vaccination trials, at least one clinical phase III study using a genetically modified whole-cell cancer vaccine had to be stopped prematurely due to inferior results in the vaccination group (48). Finally, of particular importance is the notion that in an interim analysis of the Leuven cohort children below 20 years of age (*n* = 14) obviously benefited more from DC vaccination than adults (*n* = 57) (49). However, it is too early to estimate whether this reflects a better immune response of vaccinated children or whether fundamental differences in tumor cell biology between children and adults contribute to this phenomenon. Most encouraging is the fact that DC vaccination compared favorably to standard therapy without DCs in all trials with a defined control group (35–50). In four of these trials the mean OS in the vaccinated groups was 23.9 months [15.2 (50), 31 (35), 31.0 (37), and 17.3 months (36)] compared to 10.8 months [8.6 (50), 7 (35), 15 (37), and 12.7 months (36)] in the conventional treatment group without vaccination. The fourth study, the only randomized study so far, shows a 2-year OS of 7.7% in the vaccinated group versus 0% in the non-vaccinated patients (*p* < 0.05) (38). These data also compare favorably with survival rates from standard therapy regimens with an OS of 14.6 months for primary (51) and 8.4 months for relapsed GBMs (52). However, these data must be interpreted with caution, since inclusion criteria were not identical and most studies used a historical control group only. Nevertheless, there is an unequivocal trend toward a favorable effect of DC vaccination on patient survival in all studies paired with a positive risk-benefit-assessment which justifies further pursuit of this approach.

## Design of Next-Generation DC Trials

As outlined above, for several aspects of DC vaccination there is a growing consensus (admittedly at a low level of evidence since randomized trials are still scarce). For other key issues, phase I/II clinical trials are urgently needed before phase III studies or even commercialization of a stringently produced DC vaccine can be imagined. Among these key issues the central question of the optimal maturation cocktail is not answered yet. Basically, the superiority of a cytokine-based maturation cocktail ± PGE_2_ (53) over a α-type-1 polarizing cocktail (IFNγ + TLR agonists) (43, 44, 45) or vice versa has to be shown. Since cytokine-matured DCs frequently produce no or only low-levels of IL-12, this question could be of major relevance, especially since one recent study has demonstrated a correlation between IL-12 production of DCs and time to progression (43). Furthermore, the optimal antigen source (whole tumor protein or mRNA versus peptides) should be determined. As single peptide vaccines have been shown to result in peptide-deficient escape variants (54, 55), multipeptide vaccines have recently been used with considerable success (45, 56).

Maybe the most important hindrance of immunotherapeutic approaches is the ability of high-grade glioma cells to secrete inhibitory cytokines such as TGFβ (57) and to promote accumulation and proliferation of suppressive cell populations like regulatory T cells (T_reg_). Infiltration with T_reg_ is more common and pronounced in higher-grade tumors and in astrocytic than in oligodendroglial tumors (58, 59). Although prognostically not of prime importance, infiltration with T_reg_ is associated with glioma progression (58) and has been shown to hinder successful immune responses (60, 61). Thus, there seem to be effective mechanisms in the tumor microenvironment which prevent the immune response from translating its potency into clinical efficacy. As a consequence, many attempts have been made to specifically counteract these mechanisms. Substances interfering with the TGFβ signaling pathways are currently tested in early clinical trials, e.g., as inactivating antibodies [fresolimumab (62)] or antisense oligonucleotides [trabedersen (63)]. Likewise, T_reg_-depleting strategies have been developed either as depleting antibodies (61, 64) or as metronomic, immunomodulatory chemotherapy (65, 66). Since many experimental models have shown that T_reg_-depletion modulates the tumor microenvironment (67) and permits the generation of effective antitumor responses (60, 61) these substances are attractive candidates for combination with DC vaccination.

Relapsed or refractory high-grade gliomas are extremely aggressive tumors, so that altering patient selection criteria may significantly improve DC vaccination treatment results. In fact, DC vaccination is already being included into primary glioblastoma treatment (68, 69) and one group even proceeded to vaccinate low grade glioma patients in an attempt to prevent anaplastic transformation (NCT01635283). Interestingly, patients relapsing after DC vaccination showed an increased chemosensitivity (70), arguing for an inclusion of DC vaccination into existing chemotherapy regimens.

## Concluding Remarks

Available data on DC vaccination in patients with malignant glioma allow the conclusion that this therapy is safe and feasible. Efficacy data are still of limited conclusiveness but point toward a prolonged survival in some high-risk patients and a favorable risk-benefit-assessment. Although DC vaccination in its current form is not curative in the vast majority of patients, the combination with other immunomodulatory agents, alternating chemoimmunotherapy regimens, and inclusion in treatment schedules for better-risk patients will certainly increase the number of patients who will benefit as long-term survivors from DC vaccination. Since pediatric glioma patients seem to be particularly prone to respond to DC immunotherapy, DC vaccination trials in this patient subgroup must be expedited. Despite the fact that regulatory hurdles have recently hindered the dissemination of this approach considerably, the establishment of dedicated vaccination consortiums like the HGG-Immuno network will allow a broader accessibility to this therapy and facilitate the conduct of randomized multicenter trials.

## Conflict of Interest Statement

The authors declare that the research was conducted in the absence of any commercial or financial relationships that could be construed as a potential conflict of interest.

## Supplementary Material

The Supplementary Material for this article can be found online at http://www.frontiersin.org/Pediatric_Oncology/10.3389/fped.2013.00012/abstract

Supplementary Table S1 **Design and results of clinical trials investigating the safety and efficacy of DC vaccination for the treatment of high-grade gliomas in children and adults**.
