# Supplementary material for: Dendritic Cell Vaccination in Pediatric Gliomas: Lessons Learnt and Future Perspectives
Source: Front Pediatr. 2013 Jun 10;1:12. doi: 10.3389/fped.2013.00012 (PMC3860891; doi:10.3389/fped.2013.00012)
Supplement: Supplementary Table S1 — Design and results of clinical trials investigating the safety and efficacy of DC vaccination for the treatment of high-grade gliomas in children and adults. [file 46165_Eyrich_DataSheet1.DOC]

**Supplementary online material, Table 1**. Design and results of clinical trials investigating the safety and efficacy of DC vaccination for the treatment of high grade gliomas in children and adults.

| **author** | **patients n=** | **included patients** | **DC**  **maturation** | **QC** | **release criteria** | **vaccination regimen** | **side effects** | **outcome** |
| --- | --- | --- | --- | --- | --- | --- | --- | --- |
| Liau et al., 2000 | 1 | case report  subtotal resection of relapse | GM-CSF, IL-4 (7d)+tumour-peptides over night | FACS: MHC I/II, CD80, CD86 | n.d. | 5x10^6^, biweekly | none | progress after 2nd vaccine, decompression OP, then third vaccine, DOD 8 months |
| Yu et al. , 2001 | 9 | after primary OP and irradiation, control group without DCs | GM-CSF, IL-4 (7d)+tumour-peptides over night | FACS: CD14, HLA-DR, CD86 | n.d. | 10^6^ biweekly, intradermally | 1x fever  1x LN swelling | median survival 455 days,  control group 257 days |
| Kikuchi et al. 2001 | 8 | relapse after primary treatment, detectable disease on MRI  7 adults, one 4y child | GM-CSF, IL-4, TNFα (7d)+fusion with tumour cells for 48h | FACS: MHC I/II, CD80, CD83, CD86 | n.d. | 2-8x10^6^ fDCs, every 3 weeks, 1-9 vaccines | 1x erythema | 4xSD, 4xPD  OS or TTP not given |
| Yamanaka et al. 2003 | 9 | relapse after primary treatment, no surgery before vaccination | GM-CSF, IL-4, KLH (7 d) +KLH, tumour lysate over nicht | FACS: MHC I/II, CD14, CD80, CD83, CD86 | n.d. | 1-3.2x10^7^ every 3 weeks, 1-10 vaccines intradermally, 2 patients intratumoral | 1x headache  2x erythema | 2 minor responses, 4xSD, 4xPD  OS or TTP not given |
| Yu, et al. 2004 | 14 | primary GBM after OP and irradiation,  relapsed GBM after OP,  control group without DC | GM-CSF, IL-4, KLH (7 d)  + tumour lysate over night | FACS: MHC II, CD14, CD83, CD86, CD54, CD40 | n.d. | 10^7^-10^8^ DCs, 3xbiweekly  intradermally | 3x headache  2x fatigue  1x erythema  2x seizures | DC group median survival 133 weeks,  control group 30 weeks |
| de Vlee-schouwer et al. 2004 | 1 | pediatric case report:  relapsed GBM after surgery | GM-CSF, IL-4, KLH (7 d) +IL1ß, TNFα, PGE_2_+tumour lysate for 24h | FACS: CD83 | n.d. | 1-9x10^6^, intradermally  2xbiweekly, 4xmonthly | none | still alive after >35 weeks |
| Rutkowski et al. 2004 | 12 | relapsed GBM after surgery  4 adolescents | GM-CSF, IL-4, KLH (7 d) +IL1ß, TNFa, PGE_2_+tumour lysate for 24h | FACS: MHC II, CD80, CD83, CD86 | n.d. | 2-4x10^6^, intra-dermally,  2xbiweekly, 4xmonthly | 1x brain edema  1x nocturnal sweating  1x meningeal irritation  1x morning stiffness | mean survival 10.6 months  2 long-term survivors (>35 months) |
| Kikuchi et al. 2004 | 15 | relapse after primary treatment  detectable disease | GM-CSF, IL-4, TNFα (7d)+fusion with tumour cells for 24h | FACS: fusion protein | n.d. | 3-30x10^6^ fDC, +IL-12 s.c. 3xbiweekly | 4x fever.  1x seizure  13x erythema | OS 9.8 months |
| Liau et al . 2005 | 12 | primary&relapsed GBM | GM-CSF, IL-4+tumour peptide for 1h | FACS: MHC I/II, CD14, CD40, CD80, CD83, CD86 | in DC-gate  >50% HLA-DR+CD14- | 1, 5, 10x10^6^ DC (10x10^6^ not reached in all patients), 3xbiweekly, intradermally | 4x fever  2x erythema  2x LN swelling | 2 survivors  median OS 23,4 months, TTP 15,5 months  pat. with growing tumors: OS 11,7 mo,  pat. with SD or no residual tumor: OS 35.8 mo, TTP 19.9mo  best historical control: OS 18.3 mo, TTP 8.2 mo |
| Yamanaka et al. 2005 | 24 | relapsed GBM without surgery | GM-CSF, IL-4 (7d)+KLH, tumour lysate over night | FACS: MHC I/II, CD14, CD40, CD80, CD83, CD86 | n.d. | 1-32x10^6^, intradermally, every 3 weeks, mean 7 injections | 1x headache,  6x erythema | OS 581 days  3 alive > 800 days |
| Okada et al. 2007 | 12 | primary and relapsed GBM, no progression 8 weeks after OP | GM-CSF, IL-4 (6d) +TNFα, IL1ß, IFNγ, tumour lysate (16h) | IL12p70 production after maturation | n.d. | 2x10^6^ DCs biweekly, intradermally, thigh | 1x headache  2x erythema | TTP 6 mo,  in 5 patients PD under vaccination |
| Wheeler et al. 2008 | 34 | primary&relapsed GBM after surgery | GM-CSF, IL-4 (6d)+tumour lysate for 18h | FACS: CD14, CD80, CD83, CD86, HLA-DR | >70% HLA-DR^+^CD14^-^, min. 1x10^7^ DC  gram stain,  mycoplasma, endotoxin <5IU/kg | 1-4x10^7^, i.d.  3x biweekly  1xafter 6 weeks | 1x local glioma growth after DTH testing | TTP 308 vs. 167 days in responders vs. nonresponders  OS 642 vs. 430 days in responders vs. nonresponders  chemosensitivity increased after vaccination |
| Walker et al. 2008 | 13 | primary&relapsed GBM after surgery | GM-CSF, IL-4 (6d)+monocyte conditioned medium+tumour lysate for 3d | k.A. | sterility | 10^6^ DC i.d., abdominal skin, 6xbi-weekly, then after 6 weeks | n.a. | OS 11.6 months |
| de Vlee-schouwer et al. 2008 | 56 | relapsed GBM after surgery | GM-CSF, IL-4, KLH (7 d) +IL1ß, TNFα, PGE_2_+tumour lysate for 24h | FACS: CD14, CD80, CD83, CD86, HLA-DR, CD1a, CD25 | n.d. | 6x10^6^ DCs, intradermally | 2x haematol. tox.  6x increase in focal signs  erythema in all patients | median PFS 3 months  median OS 9.6 months  better OS in younger (<35y) and totally resected patients |
| Sampson et al. 2009 | 12 | primary GBM after OP and radiotherapy | GM-CSF, IL-4, KLH (7 d) + TNFa, IL-1ß. IL-6 + EGFRvIII peptide | FACS: CD14, HLA-DR | >60% viable, <10% CD3, CD19, CD56, >60% HLA-DR and CD11c, <5 U/ml endotoxin | 3xbiweekly i.d. in upper thigh, escalating doses up to 5.9x10^7^ DCs, no DLT | No > II° | TTP 6.8 mo, OS 22.8 mo |
| Prins et al. 2011 | 23 | primary&relapsed GBM after surgery  dose cohorts with 1, 5, and 10x106DCs | GM-CSF, IL-4+tumour lysate over night | FACS:  viability, CD83, CD86, HLA-DR | >70% viable cells  >30% CD86+HLA-DR+ in DC-Gate | dose cohorts 1, 5, 10x10^6^ DCs  3xbiweekly, in case of SD second course after 3 mo | LN swelling, nausea, fever, erythema, headache, fatigue | TTP 15.9 mo, OS 31.4 mo  OS better in primary than recurrent GBM  no influence of dose (OS best in 1x10^6^ DCs, 35 vs. 29 vs. 26 mo) ,) |
| Ardon et al. 2010 | 45 | pediatric relapsed brain tumor cohort | GM-CSF, IL-4, KLH (7 d) +IL1ß, TNFα, PGE2 +tumour lysate for 24h | FACS: CD14, CD80, CD83, CD86, HLA-DR, CD1a, CD25 | n.d. | 2.8x10^6^ DC, intadermally | 5x headache  8x fatigue  3x fever  3x itching  2x vomiting  1x flu-like symptoms  erythema in all | HGG OS 13,5  GBM OS 12,2  AA OS 18.4  MB/PNET OS 5.7  Ependymoma OS 23 mo  1 alive >22mo  ATRT, n=2:  alive 34 and 53 mo |
| Okada et al. 2011 | 23 | relapsed GBM without prior surgery, HLA-A2 pos. | GM-CSF, IL-4 (6d) + IL1ß, TNFα, IFNγ, polyIC, peptide (48h) | FACS: CD86, HLA-DR | >70% CD86^+^ HLA-DR^+^ in DC-gate, gram stain, sterility, mckoplasma, endotoxin < 5 IU/kg | 1x10^7^ and 3x10^7^ DCS, 4x biweekly, intranodally axillary and inguinal LN  Poly ICLC i.m. | grade 1: fever, headache, myalgia, fatigue, flu-like symptoms | 60% immune responses  2xPR (9%)  9xSD (41%)  TTP GBM 4mo  TTP AA 13 mo |
| Fadul et al. 2011 | 10 | primary GBM after radiochemotherapy | GM-CSF, IL-4 (5d)+ TNFα. PGE_2_, tumour lysate (48h) | FACS: MHC II, CD14, CD80, CD83, CD86 | n.d. | 3x10^7^ DCs, intranodal, bilateral every 2 weeks | 1x unilateral neck pain | PFS 9.5 mo  OS 28 mo (after progress bevacizumab) |
| Chang et al. 2011 | 17 | primary and relapsed GBM after surgery  control group standard treatment without DCs (n=63) | GM-CSF, IL-4 (6d)+tumour lysate (24h) | n.a. | n.d. | mean 3.1x10^7^ DC/dose  4xweekly  2xbiweekly  4xmonthly  subcutanous above cervical LN | lymphopenia and elevated liver enzymes | OS 520 days vs. 380 in control group  5y survival 18.8% vs. 0% in control group  relapsed pat. responded better than primary GBM |
| Jie et al.  2012 | 13 (control group n=12) | primary GBM after OP+radiochemotherapy  randomized +/- DCs | GM-CSF, IL-4 (5 d), IL-1ß, PGE_2_, TNFα (24h) +heat-shocked tumour lysate | FACS: HLA-DR^+^CD11c^+^, CD86, CD83 | morphology, endotoxin | 6x10^6^ DCs intradermally, 2xweekly, 2xbiweekly | 2x fever  1x erythema | DC vs. control group  TTP 11.9 vs. 7.8 mo  2y survival 7.7 v. 0% |
| Phuphanich et al.  2012 | 16 | primary and relapsed GBM after surgery  HLA-A1 or A2 pos. | GM-CSF, IL-4 (5 d) + TNFα (3-4d)+peptides: MAGE1, AIM-2, gp100, Her2, IL13Ra2 (ICT-107) | n.a. | sterility, mycoplasma, endotoxin | intradermally in axilla, 3x every 2 weeks | fatigue, pruritus, rash | OS 27,2 mo (7/22 suviving >42 mo)  2y OS 80%, PFS 44% |
| Cho et al. 2012 | 18 (control group n=16) | primary GBM after OP+radiochemotherapy | GM-CSF, IL-4 (7 d) + irrad. tumour cells for 18-24h | FACS: MHC I+II | n.d. | 2-5x10^7^ DCs, 4xweekly, 2xbiweekly, 4xmonthly  second DC vacc.  after relapse and  reoperation | 1x transient reduced liver function  1xmild lymphopenia | Median OS 31.9 vs. 15 mo  1-, 2-, 3-y OS: 89, 44, 17% vs. 75, 19, 0%  median PFS 8.5 vs. 8 mo |
| Iwami et al. 2012 | 8 | relapsed GBM not in remission  HLA-A02 or-A24 | GM-CSF, IL-4 (6 d), IL-1ß, IL-6, TNFα (2d) +  2 IL-13Ra2 peptides | FACS: CD14, CD40, Cd80, CD83, CD86, MHC I+II | sterility, purity, viability, cell count | 1x10^7^ biweekly, max. 6 vaccines | 1xf atigue, 2x local reactions | 3x SD  4x PD  1x MR |
| Akiyama et al. 2013 | 9 | relapsed GBM without prior surgery | GM-CSF, IL-4 (6d) + TNFα, IFNγ, IFNα, polyIC, IL-1ß (48h), then 5 peptides+KLH | FACS | n.d. | 3 cohorts:  1, 2, 5x10^6^ DCs intradermally | 1x elevated liver enzymes | 8x PD  1x SD (>2y) |

*Abbreviations: AA = anaplastic astrocytoma, ATRT = atypical rhabdoid teratoid tumor, CD = cluster of differentiation, d = day, DC = dendritic cells, DLT = dose limiting toxicity, DOD= dead of disease, DTH = delayed type hyersensitivity, FACS = fluorescence-activated cell sorting, fDC = fusion dendritic cells, GBM = glioblastoma multiforme, GM-CSF = granulocyte-monocyte colony stimulating factor, h = hour, HGG = high grade glioma, HLA = human leukocyte antigen, IFN = interferon, i.d. = intradermal, IL = interleukin, IU = international units, KLH = keyhole limpet hemocyanin, LN = lymph node, MB = medulloblastoma, MHC = major histocompatibility complex, mo = month, MRI = magnetic resonance imaging, n.a. = not assessed, n.d. = not defined, OP = neurosurgical operation, OS = overall survival; PD = progressive disease; PGE_2_ = prostaglandin E_2_, PFS = progression-free survival, PNET = primitive neuroectodermal tumor, PR = partial response, QC = quality control, s.c. = subcutaneous, SD = stable disease; TNF = tumor necrosis facor, TTP = time to progression, y = year*
